# Loneliness and Perceived Stress in Older Adults Across the Cognitive Spectrum

**DOI:** 10.1002/alz70860_107148

**Published:** 2025-12-23

**Authors:** Rebecca Mullen, Lauren Gunn‐Sandell, Nichole E Carlson, Isabela Romano, Laura Gorla, Jennifer Krupa, Franklin Roberts, Bri M Bettcher

**Affiliations:** ^1^ University of Colorado Anschutz Medical Campus, Aurora, CO, USA; ^2^ University of Colorado School of Medicine, Aurora, CO, USA

## Abstract

**Background:**

Loneliness is an established risk factor for worsening health and cognition in older adults, including those with Alzheimer's disease (AD). Few studies offer the opportunity to assess loneliness and other known risk factors for poor health, like perceived stress, in older adults with and without AD. This study assessed the association between loneliness and perceived stress in a cohort of older adults across the cognitive spectrum and described differences based on symptom status.

**Method:**

We conducted a cross‐sectional assessment of asymptomatic older adults (*n* = 96) and adults symptomatic for AD (i.e., mild cognitive impairment or dementia level of severity; *n* = 40) who underwent cognitive testing and completed the Revised 20‐item UCLA Loneliness Survey and Perceived Stress Scale (PSS) (total *n* = 136). We conducted multivariable regression analysis controlling for age, sex, education, geriatric depression symptoms, and social isolation (marital status or cohabitation with partner, contact with family and friends, and participation in social organizations), and additionally explored an interaction effect.

**Results:**

The mean age of participants was 70.4 (SD 6.6), with the majority being female (*n* = 86, 63%) and White, non‐Hispanic (*n* = 126, 93%). Most participants reported moderate or high loneliness, including 66% of asymptomatic individuals (*n* = 63/96) and 53% of symptomatic individuals (*n* = 21/40). After adjusting for covariates, loneliness severity was significantly associated with symptom status (aOR: 0.92, 95% CI: 0.85‐0.98, *p* = 0.016); the odds of being considered “asymptomatic” increased by 8.5% for every 1‐point increase in loneliness score. Loneliness severity was associated with an average 0.10‐point increase in PSS; however, this was not statistically significant (95% CI: ‐0.02, 0.22, *p* = 0.10). The relationship between loneliness and PSS did not significantly differ by symptom status (95% CI: ‐0.31, 0.19, *p* = 0.63).

**Conclusion:**

We found that loneliness is common in older adults across the cognitive spectrum, and loneliness severity is significantly associated with AD symptom status, with increasing loneliness severity among asymptomatic individuals. Loneliness severity was not associated with perceived stress, potentially illustrating the influence of other contextual factors such as social support. Future studies will evaluate the relationship between loneliness severity and biomarkers in asymptomatic older adults in addition to cognitive testing across the cognitive spectrum.

